# Family Conflict and Older Chinese Americans’ Self-Efficacy in End-of-Life Care Planning: The Role of Acculturation

**DOI:** 10.1093/geroni/igab046.2188

**Published:** 2021-12-17

**Authors:** Kaipeng Wang, Fei Sun, Yanqin Liu, Carson De Fries

**Affiliations:** 1 University of Denver, Denver, Colorado, United States; 2 Michigan State University, East Lansing, Michigan, United States; 3 Mayo Clinic Arizona, Scottsdale, Arizona, United States

## Abstract

Family involvement in end-of-life (EOL) care is critical to ensure older adults’ health and quality of life. Older adults’ self-efficacy in discussing EOL care plans with family members can facilitate family involvement in EOL care planning. Research shows that family relationships are associated with self-efficacy in discussing EOL care with family members among older Chinese Americans. However, the roles of family conflict and acculturation remain unknown. This study examines the association between family conflict and self-efficacy in discussing EOL care with family members and whether such an association differs by acculturation levels among older Chinese Americans. Data were collected from 207 Chinese Americans aged 65-102 in two metropolitan areas in 2017. Ordinary least squares regression was conducted to examine the association between family conflict, acculturation, and self-efficacy in discussing EOL care with family. Family conflict was negatively associated with older adults’ self-efficacy in discussing EOL care with family. More specifically, the negative association between family conflict and self-efficacy in discussing EOL care with family members was more pronounced for those with higher levels of acculturation. Findings highlighted differential effects of family conflict on self-efficacy of EOL care plan discussion for older adults with different acculturation levels. Those with higher acculturation may be more independent in their EOL care planning and aware of the possible negative effects of family conflict in their EOL care planning discussions. Acculturation needs to be considered by geriatric health providers to develop family-centered interventions in improving end-of-life care planning for this population.

